# Supplementary material for: Potential SARS-CoV-2 Susceptibility of Cetaceans Stranded along the Italian Coastline
Source: Pathogens. 2022 Sep 25;11(10):1096. doi: 10.3390/pathogens11101096 (PMC9607105; doi:10.3390/pathogens11101096)
Supplement: Supplementary file 1 [file pathogens-11-01096-s001.zip › pathogens-1899279-supplementary.pdf]

Supplementary material

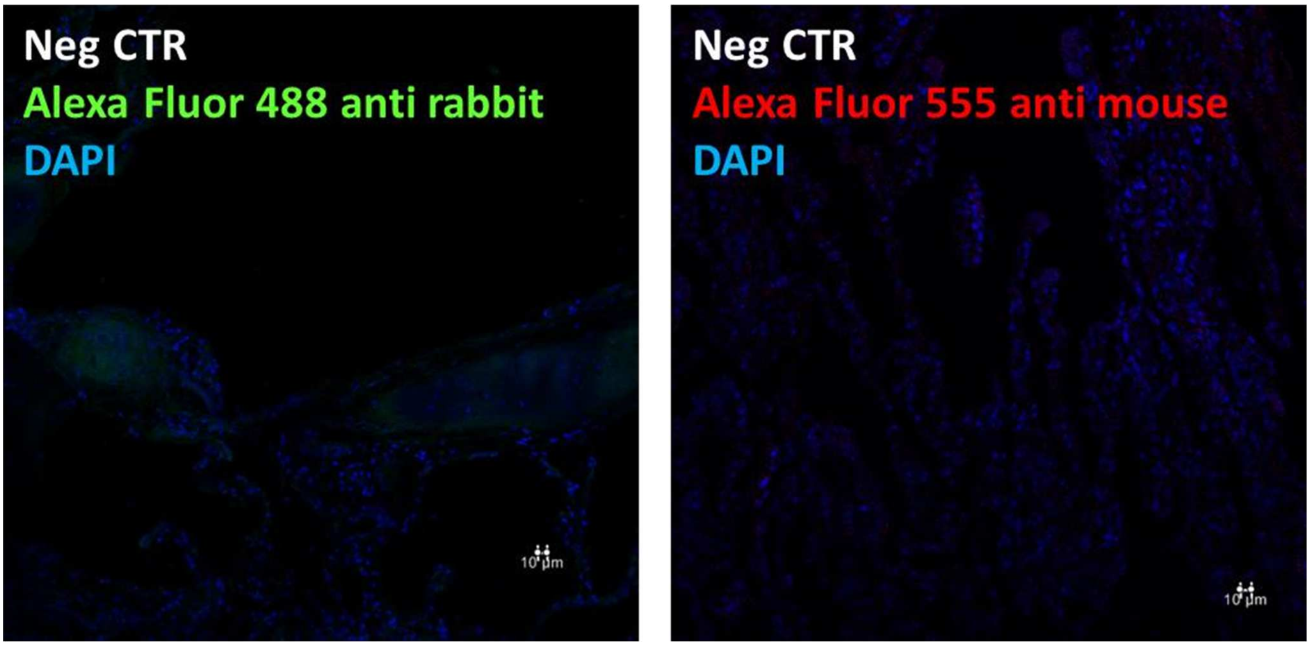

Figure S1. Immunofluorescence of negative control (Neg CTR) in lungs from *T. truncates* and with donkey Alexa 488 or Alexa 555 conjugated secondary antibodies. DAPI (blu):4,6-diamidino-2-phenylindole. Scale bars, 10 µm.

Table S2. Samples tested for SARS-CoV 2 by IHC

Legend

ID (case IHC) reference number of lung specimens analyzed by IHC (some also underwent PCR; *specimens that underwent only IHC; Latitude/longitude refer to the geolocation coordinates of stranding sites

Year	ID (case IHC)	SPECIE	Long.	Lat.
2020	1	*T.truncatus*	9.10342	44.18731
	2	*S.coeruleoalba*	8.27576	44.17631
	3	*S.coeruleoalba*	8.27154	44.17352
	4	*S.coeruleoalba*	8.24996	44.12117
	5	*Z. cavirostris*	7.4602	43.4872
	6	*S.coeruleoalba*	9.2346	44.1626
	7	*S.coeruleoalba*	8.26881	44.15035
	8	*S.coeruleoalba*	8.26057	39.16965
	9	*S.coeruleoalba*	9.40432	39.45169
	10	*T.truncatus*	9.31353	41.2195

	11	*S.coeruleoalba*	8.2784	39.348
	12	*T.truncatus*	9.3108	41.1517
	13	*T.truncatus*	8.18597	40.34847
	14	*S.coeruleoalba*	8.26279	44.12455
	15	*S.coeruleoalba*	12.4069	37.33581
	16	*G. griseus*	15.1422	38.1386
	17	*T.truncatus*	12.4134	37.3372
	18	*S.coeruleoalba*	12.55937	38.1696
	19	*T.truncatus*	13.14505	38.114
	20	*S.coeruleoalba*	13.18666	37.22627
	21	*S.coeruleoalba*	15.1818	37.246
	22	*S.coeruleoalba*	13.33246	38.2933
	23	*S.coeruleoalba*	12.2552	37.4815
	24	*S.coeruleoalba*	14.58892	38.9023
	44*	*S.coeruleoalba*	15.92121	38.68673
	45*	*S.coeruleoalba*	14.3924	40.47324
	46*	*S.coeruleoalba*	13.51668	40.43214
	47*	*S.coeruleoalba*	14.42146	40.38789
	48*	*S.coeruleoalba*	15.10666	38.9336
	49*	*S.coeruleoalba*	13.24238	38.6017
	50*	*S.coeruleoalba*	10.1038	42.4404
	51*	*S.coeruleoalba*	10.13428	43.53571
	52*	*S.coeruleoalba*	12.83074	41.41406
	53*	*S.coeruleoalba*	10.26434	43.22239
	54*	*S.coeruleoalba*	12.18349	41.43059
	55*	*T.truncatus*	11.47904	42.5196
	56*	*Balaenoptera physalus*	14.225	40.3774
	57*	*S.coeruleoalba*	15.19819	40.1076
	58*	*S.coeruleoalba*	15.33574	40.4552
	59*	*T.truncatus*	12.61943	41.44484
2021	25	*S.coeruleoalba*	9.1345	44.20416

	26	*S.coeruleoalba*	7.36784	43.47191
	27	*S.coeruleoalba*	8.689024	44.39423
	28	*T.truncatus*	9.102	44.186
	29	*S.coeruleoalba*	13.24238	38.6017
	30	*S.coeruleoalba*	12.5584	38.1676
	31	*S.coeruleoalba*	12.55936	38.1695
	32	*S.coeruleoalba*	13.32524	38.4638
	33	*D. delphis*	12.4188	37.3384
	34	*S.coeruleoalba*	15.30515	38.17411
	35	*S.coeruleoalba*	12.78526	37.58016
	36	*S.coeruleoalba*	13.51327	37.59333
	37	*T.truncatus*	8.28059	44.17848
	38	*T.truncatus*	9.25493	44.15468
	39	*T.truncatus*	8.923392	44.41023
	40	*S.coeruleoalba*	9.6232	44.22234
	41	*T.truncatus*	8.8383	43.56767
2022	42	*T.truncatus*	8.029215	43.88192
	43	*T.truncatus*	8.125	43.52574

Table S3. ACE2 Immunohistochemical analysis of lung tissue from the Mediterranean Marine Mammal Tissue Bank (MMMTB), University of Padua (Legnaro, Padua, Italy).

ID	Species	Sex	Age	Origin	IHC ACE-2
2	*T.truncatus*	Female	Adult	Wild	-
20	*T.truncatus*	Female	Adult	Captivity	++
123	*T.truncatus*	Female	Calf	Captivity	++
133	*T.truncatus*	Female	Adult	Captivity	++
139	*T.truncatus*	Male	Adult	Captivity	+
142	*T.truncatus*	Female	Adult	Wild	+
144	*T.truncatus*	Male	Calf	Captivity	+
145	*T.truncatus*	Male	Calf	Captivity	++
162	*T.truncatus*	Male	Calf	Wild	++
190	*T.truncatus*	Male	Adult	Wild	+
201	*T.truncatus*	Male	Adult	Wild	-
312	*T.truncatus*	Female	Juvenile	Wild	++
343	*T.truncatus*	Female	Calf	Captivity	++
349	*T.truncatus*	Female	Juvenile	Wild	++
359	*T.Truncatus*	Female	Calf	Captivity	-
493	*T.truncatus*	Male	Adult	Captivity	++

159	*T.truncatus*	Male	Adult	Captivity	+
6	*S.coeruleoalba*	Male	Adult	Wild	-
45	*S.coeruleoalba*	Female	Adult	Wild	-
167	*S.coeruleoalba*	Male	Adult	Wild	++
170	*S.coeruleoalba*	Female	Adult	Wild	++
255	*S.coeruleoalba*	Female	Juvenile	Wild	++
263	*S.coeruleoalba*	Female	Adult	Wild	++
322	*S.coeruleoalba*	Female	Juvenile	Wild	-
341	*S.coeruleoalba*	Male	Juvenile	Wild	-
353	*S.coeruleoalba*	Female	Adult	Wild	+
355	*S.coeruleoalba*	Female	Juvenile	Wild	-
374	*S.coeruleoalba*	Female	Calf	Wild	-
394	*S.coeruleoalba*	Male	Juvenile	Wild	-
447	*S.coeruleoalba*	Male	Adult	Wild	-
454	*S.coeruleoalba*	Male	Juvenile	Wild	+
479	*S.coeruleoalba*	Male	Calf	Wild	++

Legend of Results of ACE2 expression by IHC: ++ Highly expressing sample; + Weakly expressing sample; - Negative sample.

Sample analysed also by double IF

Table S1. ACE2 Immunohistochemical analysis of lung tissue from the Mediterranean Marine Mammal Tissue Bank (MMMTB), University of Padua (Legnaro, Padua, Italy).

ID	Species	Sex	Age	Origin	IHC ACE-2
2	*T.truncatus*	Female	Adult	Wild	-
20	*T.truncatus*	Female	Adult	Captivity	++
123	*T.truncatus*	Female	Calf	Captivity	++
133	*T.truncatus*	Female	Adult	Captivity	++
139	*T.truncatus*	Male	Adult	Captivity	+
142	*T.truncatus*	Female	Adult	Wild	+
144	*T.truncatus*	Male	Calf	Captivity	+
145	*T.truncatus*	Male	Calf	Captivity	++
162	*T.truncatus*	Male	Calf	Wild	++
190	*T.truncatus*	Male	Adult	Wild	+
201	*T.truncatus*	Male	Adult	Wild	-
312	*T.truncatus*	Female	Juvenile	Wild	++
343	*T.truncatus*	Female	Calf	Captivity	++
349	*T.truncatus*	Female	Juvenile	Wild	++
359	*T.Truncatus*	Female	Calf	Captivity	-
493	*T.truncatus*	Male	Adult	Captivity	++
159	*T.truncatus*	Male	Adult	Captivity	+
6	*S.coeruleoalba*	Male	Adult	Wild	-
45	*S.coeruleoalba*	Female	Adult	Wild	-
167	*S.coeruleoalba*	Male	Adult	Wild	++
170	*S.coeruleoalba*	Female	Adult	Wild	++
255	*S.coeruleoalba*	Female	Juvenile	Wild	++
263	*S.coeruleoalba*	Female	Adult	Wild	++
322	*S.coeruleoalba*	Female	Juvenile	Wild	-
341	*S.coeruleoalba*	Male	Juvenile	Wild	-

353	*S.coeruleoalba*	Female	Adult	Wild	+
355	*S.coeruleoalba*	Female	Juvenile	Wild	-
374	*S.coeruleoalba*	Female	Calf	Wild	-
394	*S.coeruleoalba*	Male	Juvenile	Wild	-
447	*S.coeruleoalba*	Male	Adult	Wild	-
454	*S.coeruleoalba*	Male	Juvenile	Wild	+
479	*S.coeruleoalba*	Male	Calf	Wild	++

Legend of Results of ACE2 expression by IHC: ++ Highly expressing sample; + Weakly expressing sample; - Negative sample.

Sample analysed also by double IF
